# Simple Schizophrenia or Neurotic Disorder? Case report

**DOI:** 10.1192/j.eurpsy.2022.1140

**Published:** 2022-09-01

**Authors:** P. Suz Maroto, B. Díez Valle, A. Hurtado De Mendoza Vázquez, E. Navas Collado

**Affiliations:** 1 Hospital Universitario José Germain, Psychiatry, Leganés, Spain; 2 Hospital Severo Ochoa, Psychiatry, Leganés, Spain

**Keywords:** psychiatric classifications, simple schizophrenia, diagnosis

## Abstract

**Introduction:**

The diagnosis of simple schizophrenia remains an unusual and controversial diagnosis today. The presentation of nonspecific symptoms shared by other nosological entities make differential diagnosis a challenge.

**Objectives:**

The main objective of this case report is to review the diagnosis of simple schizophrenia and its differential diagnosis.

**Methods:**

Case report and literature review. We present the case of a 52-year-old man who was admitted to a medium stay unit for psychosocial rehabilitation with the diagnosis of simple schizophrenia after his debut at 49 years of age with clinical manifestations of progressive self-care abandonment and personality change.

**Results:**

Given the psychosocial deterioration observed and lack of response to pharmacological and psychotherapeutic treatments, the possible diagnoses of dementia praecox and simple schizophrenia were considered. Several individual and family interviews, neuropsychological and projective tests (HTP test, figure 1-3) were performed in order to define the diagnosis. The results revealed age-appropriate cognitive functioning and the absence of data suggestive of an underlying psychotic disorder. On the other hand, it was observed that the patient was able to establish some social relationships and participate in group activities in the medium stay unit. These findings suggest the influence of factors related to the socio-familial environment and cast doubt on the initial diagnostic hypothesis.

**Figure:**
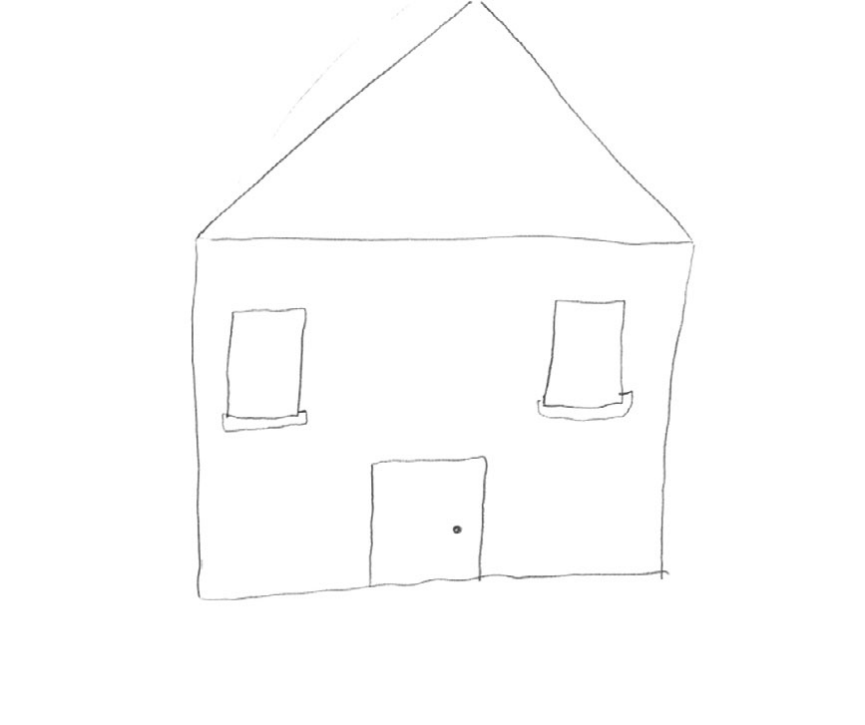

**Figure:**
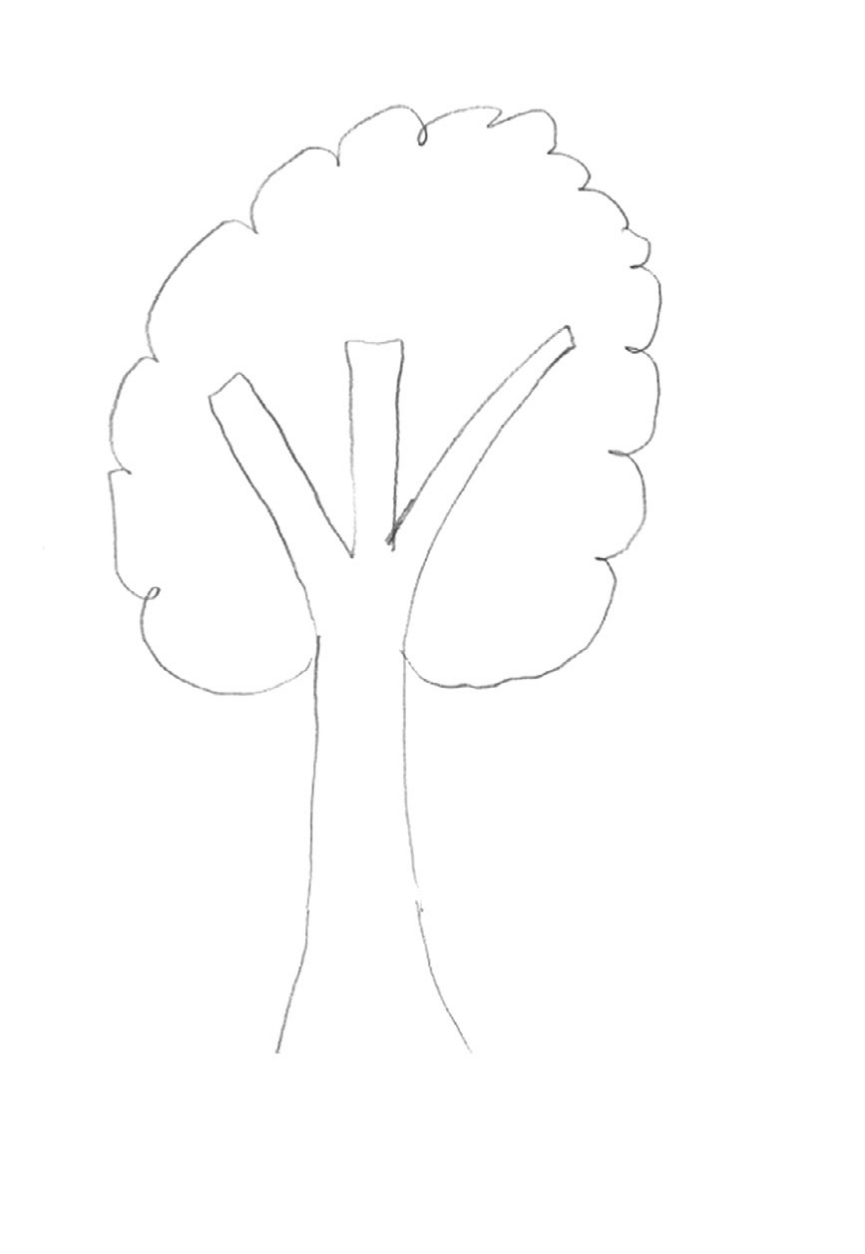

**Figure:**
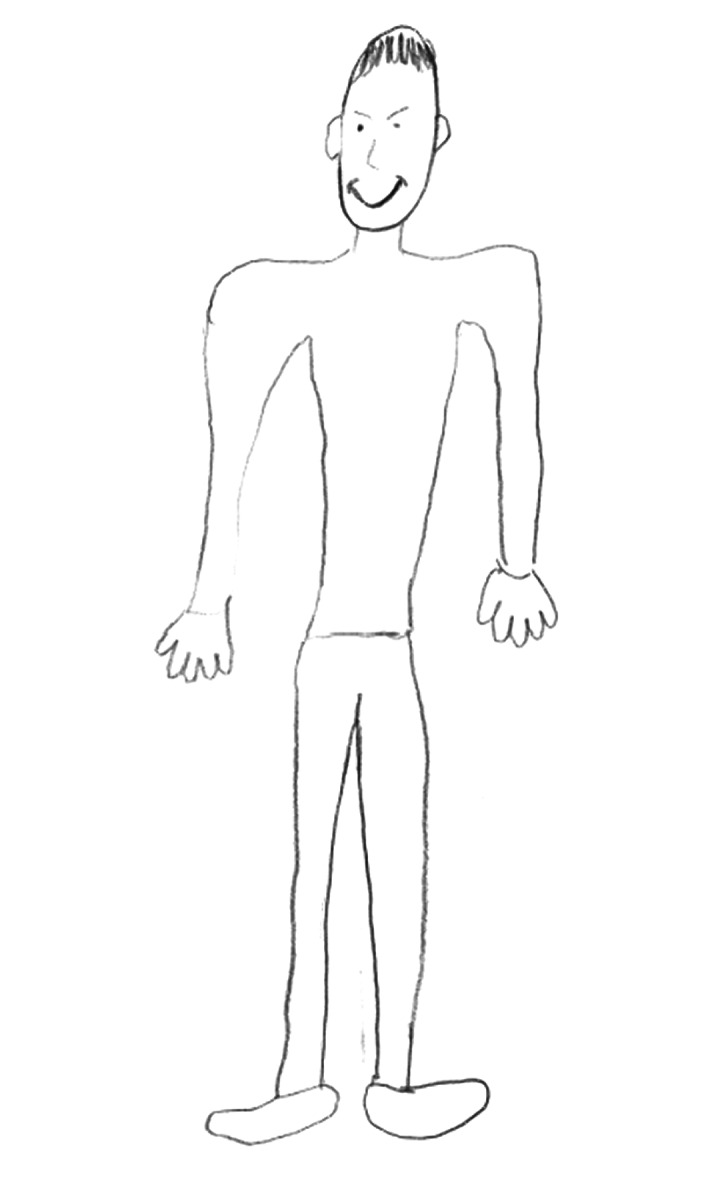

**Conclusions:**

The diagnosis of simple schizophrenia continues to present itself as a complex diagnosis that requires a careful review of the differential diagnosis.

**Disclosure:**

No significant relationships.

